# Unsatisfactory risk factor control and high rate of new cardiovascular events in patients with myocardial infarction and prior coronary artery disease

**DOI:** 10.1186/s12872-019-1062-y

**Published:** 2019-03-28

**Authors:** Jarle Jortveit, Sigrun Halvorsen, Anete Kaldal, Are Hugo Pripp, Ragna Elise S. Govatsmark, Jørund Langørgen

**Affiliations:** 1 Department of Cardiology, Sørlandet Hospital, Box 783, Stoa, 4809 Arendal, Norway; 2 Department of Cardiology, Oslo University Hospital and University of Oslo, Oslo, Norway; 3 Oslo Centre of Biostatistics and Epidemiology, Oslo University Hospital, Oslo, Norway; 4 Department of Medical Quality Registers, St. Olav’s Hospital, Trondheim, Norway; 5 Department of Heart Diseases, Haukeland University Hospital, Bergen, Norway

**Keywords:** Secondary prevention, Myocardial infarction, Risk factors

## Abstract

**Background:**

Patients with established coronary artery disease (CAD) have an increased risk of new cardiovascular events. An underuse of secondary preventive drugs has been observed, and many patients may not attain the treatment goals for secondary prevention. The aims of the present nationwide register-based cohort study were to assess the degree of risk factor control and long-term outcomes in patients < 80 years with Type 1 myocardial infarction (MI) with and without prior CAD.

**Methods:**

Data concerning all patients with MI admitted to hospitals in Norway from 2013 to 2016 were retrieved from the *Norwegian Myocardial Infarction Register* (NORMI). Long-term mortality was obtained through linkage with the *Norwegian Cause of Death Registry*.

**Results:**

In total, 47,204 patients were registered in the NORMI from 2013 to 2016. Prior CAD was recorded in 7219 (25.2%) of the 28,607 patients < 80 years old with Type 1 MIs. On average, 3 of the 6 defined treatment targets for secondary preventive therapy were attained, and only 1% of the patients achieved all targets. Patients with MI and prior CAD had increased risk of death or new MI compared to patients without prior CAD during long-term follow-up (adjusted HR 1.6, 95% CI 1.5–1.7).

**Conclusions:**

Prior CAD was frequent in patients with acute MI. The attainment of secondary preventive treatment targets in patients with MI and prior CAD was not optimal, and the long-term outcomes were reduced compared to patients without prior CAD. Increased efforts to improve risk factor control are needed.

## Background

Ischaemic heart disease remains a frequent cause of mortality and morbidity in Europe [1]. Patients with established coronary artery disease (CAD), defined as prior myocardial infarction (MI), percutaneous coronary intervention (PCI) or coronary artery bypass grafting (CABG), have a high risk of new cardiovascular events or death [2]. All patients with diagnosed CAD should be given advises regarding therapeutic lifestyle changes, including physical activity, dietary modification/weight loss, and smoking cessation, and administered adjunctive drug therapies of proven benefit in reducing the risk of new events [3–8]. Despite extensive documentation and recommendations in international guidelines, an underuse of secondary preventive drugs has been observed following MI [9]. The EUROpean Action on Secondary and Primary prevention by Intervention to Reduce Events (EUROASPIRE), the REduction of Atherothrombosis for Continued Health (REACH) and the prospeCtive observational LongitudinAl RegIstry oF patients with stable coronary arterY disease (CLARIFY) demonstrated that many patients with CAD did not attain the treatment goals for secondary prevention [10–13]. Similar findings have also been reported by local hospitals in Norway and from other European countries [14, 15]. In previous studies, the risk of selection bias and low response rates were matters of concern and limited the validity of the findings. In Norway, national health registers with mandatory registration by law and almost complete follow-up provide an opportunity to study a nationwide and unselected patient population.

The aims of the present study were to investigate the degree of risk factor control in a nationwide cohort of all patients admitted to hospitals in Norway with acute MI and prior CAD, and to study long-term outcomes in these patients compared to patients with acute MI but without prior CAD.

## Methods

### Study population

All patients < 80 years admitted to Norwegian hospitals with Type 1 MI from 1 January 2013, to 31 December 2016, and registered in the *Norwegian Myocardial Infarction Register* (NORMI) were included in this study. Registration in this registry is mandatory (the Norwegian Cardiovascular Disease Registry Regulation and the Norwegian Health Register Act), and consent by the patient is not required. The registry contains information on gender, age, known risk factors, previous illness and medication, symptoms and clinical findings on admission, in-hospital assessment, therapy and complications, drugs prescribed at discharge, and time of death. The registration and quality of the information in NORMI have been described previously [16–18]. The index MI was defined as the first Type 1 MI in the study period.

### Definitions

#### Myocardial infarction

The NORMI adhered to the third universal definition of myocardial infarction in the study period [19]. Troponin was the preferred biochemical marker of MI. A diagnostic cut point of ≥30 ng/l for troponin T was used up to May 2013. After 1 June 2013, the diagnostic limit of troponin T > 14 ng/l was recommended. The reference limits (99th percentile) for troponin I were dependent on the manufacturer. All MIs were classified as ST-elevation MI (STEMI) or non-ST-elevation MI (nSTEMI) on the basis of the diagnostic ECG [19].

#### Secondary preventive treatment targets

In accordance with the European Society of Cardiology guidelines, the following secondary preventive treatment targets were defined: [3, 4, 7, 8].

- Daily use of acetylsalicylic acid
- Daily use of statins
- No smoking
- Blood pressure < 140/90 mmHg
- LDL cholesterol < 1.8 mmol/L
- Body mass index (BMI) < 25 kg/m^2^
- HbA1c ≤7.0% in patients with diabetes

### Outcomes and follow-up

The primary endpoint was a composite of all-cause mortality or new MI during follow-up. The secondary endpoint was all-cause mortality during follow-up. The NORMI were linked to the *Norwegian Cause of Death Registry* to obtain the number of deaths and death dates. New MIs were obtained from the NORMI. Follow-up with respect to new MIs was complete until 31 December 2016, and until 31 December 2017, with respect to death.

### Statistical analysis

Continuous variables are presented as the mean ± SD (standard deviation) or median (25th percentile, 75th percentile), and differences between groups were analysed using independent sample T tests or Mann-Whitney non-parametric tests or, as appropriate. Categorical variables are presented as numbers and percentages, and differences between groups were analysed by the chi-squared test. Odds ratios (OR) were calculated by logistic regression and were age- and gender-adjusted. Kaplan-Meier curves for event-free survival were estimated and Cox regression analyses were used to calculate hazard ratios (HRs) with 95% confidence intervals (CIs) for death and/or new MI. The following covariates were included in the multivariate analyses: age, gender, smoking, history of heart failure, diabetes, hypertension, renal failure, coronary angiogram and relevant medication (acetylic acid, dual antiplatelet therapy and statins). A *p*-value of < 0.05 was regarded as statistically significant. The data were analysed using the programme STATA, version 15 (StataCorp LLC, College Station, TX, USA).

### Ethics

The Regional Committee for Medical and Health Research Ethics approved the study (REK 2016/170).

## Results

**Fig. 1:**
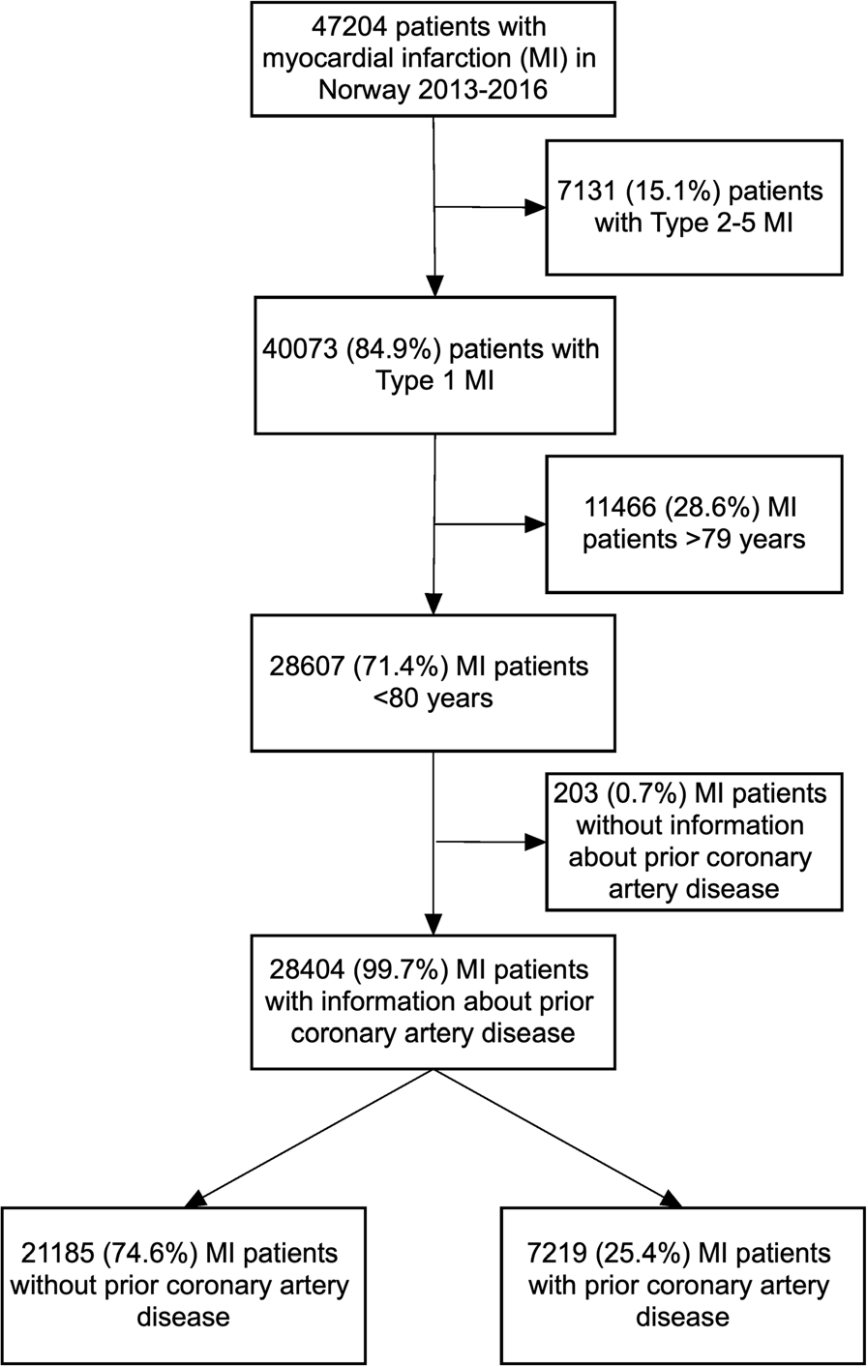
Patients admitted to hospitals in Norway with myocardial infarction from 2013 to 2016

During the period 2013–2016, 47,204 patients were registered in the NORMI. Of these, 28,607 were < 80 years old and diagnosed with Type 1 MI (Fig. 1). A history of CAD was recorded in 7219 (25.2%) patients. The clinical characteristics at the time of the index MI in patients with and without prior CAD are presented in Table 1. Previous MI was the most frequent type of prior CAD. Patients with prior CAD were older and more likely to have hypertension and diabetes compared to patients without prior CAD. They were also more likely to have quit smoking.

**Table 1:** Clinical characteristics of patients < 80 years with Type 1 myocardial infarction, Norway 2013–2016 (*n* = 28,404)

	Prior coronary artery disease		No prior coronary artery disease		*p*
	*n* = 7219		*n* = 21,185		
		Missing data		Missing data	
Male (n (%))	5785 (80)	0 (0)	15,277 (72)	0 (0)	< 0.001
Mean age (years (SD))	67 (10)	0 (0)	62 (10)	0 (0)	< 0.001
Prior coronary artery disease (n (%))					
Myocardial infarction	5638 (78)	29 (0.4)			
Percutaneous coronary intervention	5072 (70)	58 (0.8)			
Coronary artery bypass grafting	2228 (31)	9 (0.1)			
Smoking (n (%))		408 (6)		1048 [5]	< 0.001
Never smoked	1417 [20]		5339 (25)		< 0.001
Ex-smoker	3067 (43)		6290 (30)		< 0.001
Smoker	2327 (32)		8508 (40)		< 0.001
Antihypertensive treatment (n (%))	4262 (59)	43 (0.6)	8055 (38)	38 (0.2)	< 0.001
Diabetes (n (%))	2169 (30)	8 (0.1)	3052 (14)	13 (0.1)	< 0.001
Medication prior to admission (n (%))					
Acetylsalicylic acid	5753 (80)	95 (1)	3668 (17)	176 (1)	< 0.001
Statins	5353 (74)	110 (2)	4013 (19)	186 (1)	< 0.001
Beta blockers	4720 (65)	131 (2)	2888 [14]	263 (1)	< 0.001
ACE/AII receptor inhibitors	3599 (50)	110 (2)	5474 (26)	227 (1)	< 0.001
Diuretics	1897 [26]	110 (2)	2752 [13]	206 (1)	< 0.001
ST-elevation myocardial infarction (n (%))	1395 [19]	235 (3)	8185 (39)	845 (4)	< 0.001
Median systolic blood pressure (mmHg (25th, 75th percentile))	140 (123, 159)	251 (4)	140 (124, 160)	688 (3)	0.02
Median diastolic blood pressure (mmHg (25th, 75th percentile))	80 (70, 90)	251 (4)	82 (72, 93)	688 (3)	< 0.001
Median HbA1c (% (25th, 75th percentile)) if diabetes	7.5 (6.6, 8.5)	1116 (52)	7.4 (6.6, 8.7)	1493 (49)	0.74
Median total cholesterol (mmol/L (25th, 75th percentile))	4.1 (3.5, 5.0)	1809 [25]	5.2 (4.4, 6.0)	4534 (21)	< 0.001
Median LDL cholesterol (mmol/L (25th, 75th percentile))	2.3 (1.7, 3.0)	2790 (39)	3.4 (2.6, 4.1)	7441 (35)	< 0.001
Median body mass index (kg/m^2^ (25th, 75th percentile)	27.3 (24.7, 30.2)	1323 [18]	26.9 (24.5, 29.9)	3265 (15)	0.01

Patients with MIs and prior CAD were less likely to undergo coronary angiography and PCI and less likely to be prescribed secondary preventive therapy at their discharge from hospital compared to patients with no prior CAD (Table 2).

**Table 2:** Treatment of patients < 80 years with Type 1 myocardial infarction (*n* = 28,404), Norway 2013–2016

	Prior coronary artery disease		No prior coronary artery disease			
	*n* = 7219		*n* = 21,185			
		Missing data		Missing data		
	n (%)	n (%)	n (%)	n (%)	Odds ratio (95% CI, *p*)^1)^	Age- and gender-adjusted odds ratio (95% CI, *p*)^1)^
Coronary angiogram	5951 (82)	2 (0)	18,873 (89)	0 (0)	0.6 (0.5–0.6, < 0.001)	0.6 (0.6–0.7 < 0.001)
Percutaneous coronary intervention (PCI)	4179 (58)	0 (0)	15,178 (72)	1 (0)	0.6 (0.6–0.7, < 0.001)	0.6 (0.6–0.7, < 0.001)
Medication at discharge (discharged alive, *n* = 27,442)						
Acetylsalicylic acid	6682 (96)	5 (0)	19,907 (97)	20 (0)	0.8 (0.7–0.9, < 0.001)	0.9 (0.7–1.0, 0.03)
Dual antiplatelet therapy (DAPT)	5949 (86)	12 (0)	18,228 (89)	32 (0)	0.8 (0.7–0.8, < 0.001)	0.9 (0.8–0.9, 0.001)
Statins	6348 (92)	7 (0)	18,985 (93)	26 (0)	0.9 (0.8–0.9, 0.003)	0.9 (0.8–1.0, 0.06)

^1)^ Reference: No prior coronary artery disease

**Fig. 2:**
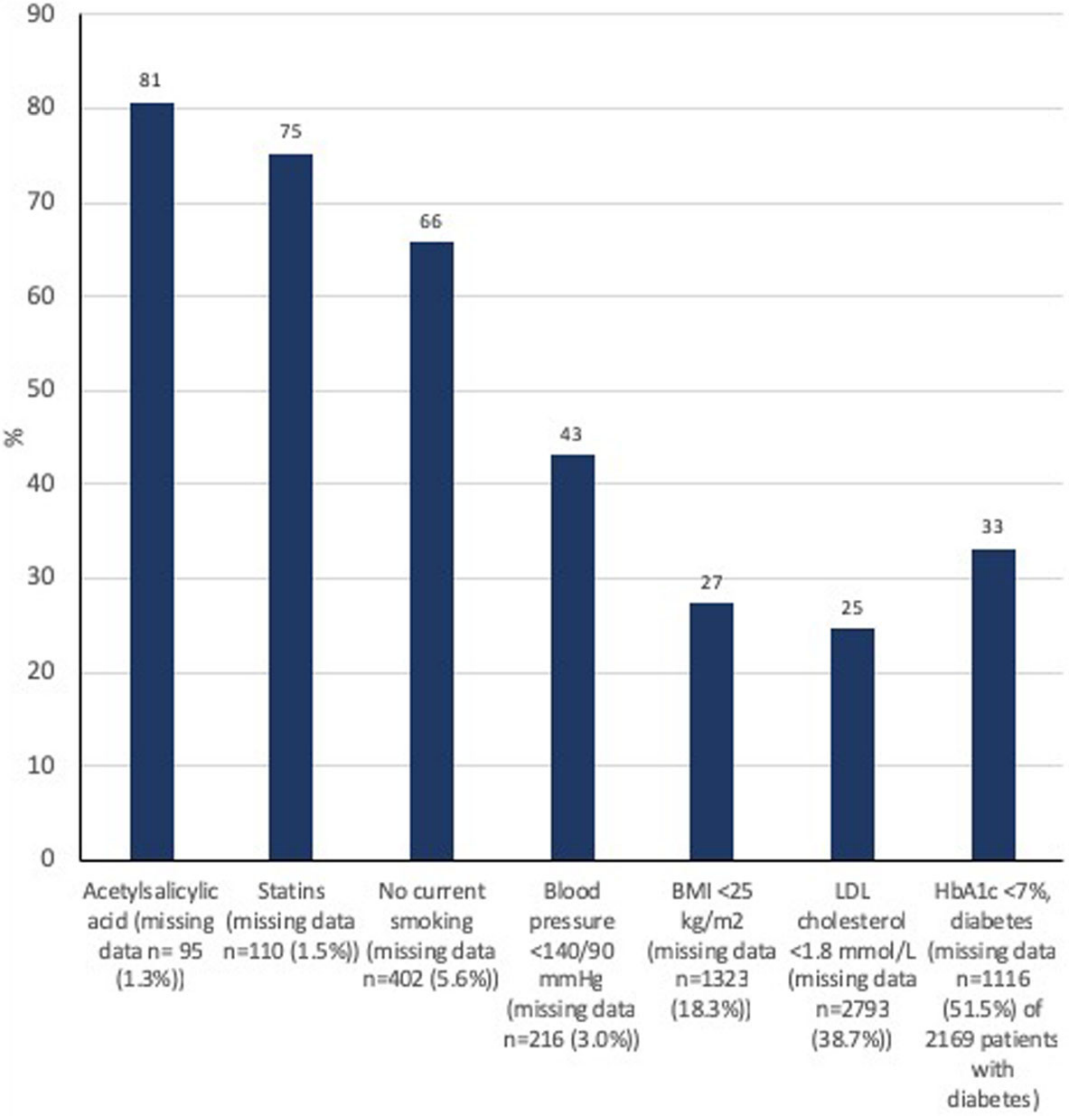
Risk factor control in patients < 80 years with coronary artery disease hospitalized with Type 1 myocardial infarction in Norway from 2013 to 2016 (*n* = 7219) (Patients with missing data were excluded)

The attainment of secondary preventive treatment goals in patients < 80 years with prior CAD is shown in Fig. 2. The risk factor control was low. About one out of five did not use acetylsalicylic acid and statins. One out of three smoked despite prior CAD. Only 43% of patients had satisfactory blood pressure control. Unfortunately, the NORMI did not have complete coverage with respect to BMI, LDL cholesterol and HbA1c. In patients with recorded information, the percentage achieving treatment targets was 27% for BMI (1611 of 5896 patients), 25% for LDL cholesterol (1092 of 4426 patients), and 33% for HbA1c in patients with diabetes (349 of 1053 patients).

**Fig. 3:**
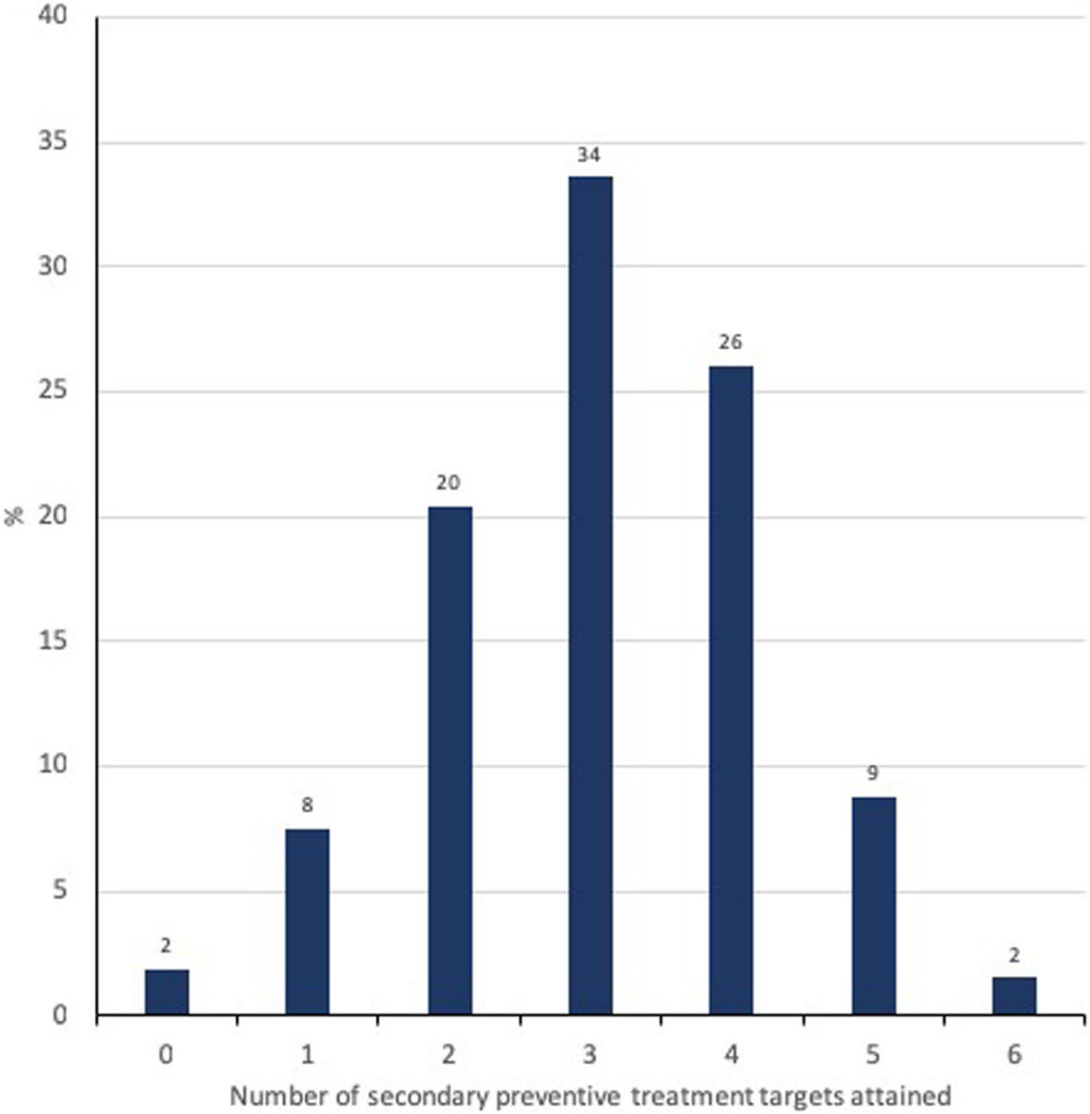
The numbers of secondary preventive treatment targets* attained in patients < 80 years hospitalized with Type 1 myocardial infarction and having prior coronary artery disease in Norway from 2013 to 2016 (n = 7219) (No smoking, blood pressure < 149/90 mmHg, LDL cholesterol < 1.8 mmol/L, body mass index < 25 kg/m^2^, acetylsalicylic acid and statin use.) (Missing information was counted as “not attained”)

The mean number of treatment targets achieved was three (Fig. 3). Only 72 (1.0%) patients with prior CAD were registered as having achieved all secondary preventive treatment targets at hospital admission. We found no differences in the overall achievement of treatment targets between different age groups (data not shown). Women were less likely to smoke (age-adjusted OR 0.8, 95% CI 0.7–0.9, *p* = 0.001) and to be overweight (age-adjusted OR 0.7, 95% CI 0.6–0.8, *p* < 0.001) compared to men. We found no gender differences for the other risk factors, but women used less statins than men (age-adjusted OR 0.8, 95% CI 0.7–0.9, p < 0.001).

**Fig. 4:**
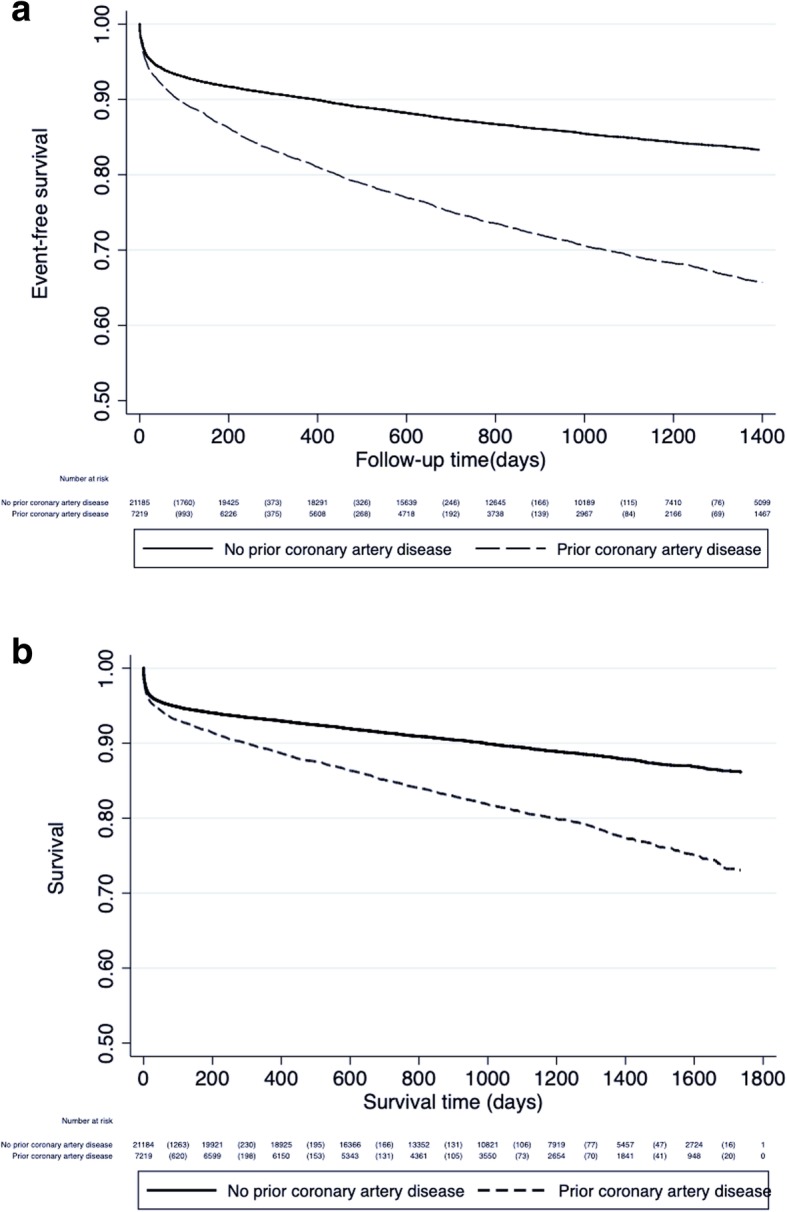
a Death or recurrent myocardial infarction during long-term follow-up in patients < 80 years with Type 1 myocardial infarction, with and without prior coronary artery disease in Norway from 2013 to 2016. b Cumulative survival following Type 1 myocardial infarction in patients < 80 years with and without prior coronary artery disease in Norway from 2013 to 2016

In total, 2162 (30.0%) and 3118 (14.7%) patients with and without prior CAD, experienced a new MI and/or death (Median follow-up time: 944 days (25th, 75th percentile: 548, 1218)).

and 1411 (19.6%) patients with and 2232 (10.5%) patients without prior CAD died (median follow-up time 1004 days (25th, 75th percentile: 609, 1400)) during the follow-up time (Fig. 4). The risk of recurrent MI or death during the study period was significantly increased in patients with prior CAD, also after multivariate adjustment (Table 3).

**Table 3:** Long-term outcomes in patients < 80 years hospitalized with Type 1 myocardial infarction, Norway 2013–2016

	Prior coronary artery disease	No prior coronary artery disease				
	*n* = 7219	*n* = 21,185				
	n (%)	n (%)	Unadjusted HR (95% CI, *p*)^1)^	Age- and gender adjusted HR (95% CI, *p*)^1)^	Multivariate adjusted HR - model 1^4)^(95% CI, *p*)^1)^	Multivariate adjusted HR - model 2^5)^(95% CI, *p*)^1)^
Myocardial infarction or death^2)^	2162 (30.0)	3118 (14.7)	2.2 (2.1–2.3, < 0.001)	1.8 (1.7–1.9, < 0.001)	1.7 (1.6–1.7, < 0.001)	1.6 (1.5–1.7, < 0.001)
Death^3)^	1411 (19.6)	2232 (10.5)	1.9 (1.8–2.0, < 0.001)	1.5 (1.4–1.6, < 0.001)	1.3 (1.2–1.4, < 0.001)	1.3 (1.2–1.4, < 0.001)

^1)^ Reference: No prior coronary artery disease

^2)^ Median follow-up time: 944 days (25th, 75th percentile: 548, 1218)

^3)^ Median follow-up time 1004 days (25th, 75th percentile: 609, 1400)

^4)^ Age, gender, diabetes, smoking, hypertension, heart failure and renal failure

^5)^ Age, gender, diabetes, smoking, hypertension, heart failure, renal failure, coronary angiogram and medication at discharge (acetylic acid, dual antiplatelet therapy and statins)

High adherence to secondary preventive treatment was associated with improved outcome. If ≥3 secondary preventive treatment targets were attained, the risk of recurrent MI or death was significantly lower compared to patients with lower degrees of risk factor control (Table 4).

**Table 4:** Risk of recurrent myocardial infarction or death according to attainment of preventive treatment targets in patients < 80 years with Type 1 myocardial infarction and prior coronary artery disease, Norway 2013–2016

	0–2 preventive treatment targets attained	3–6 preventive treatment targets attained			
	*n* = 2220	*n* = 4999			
	n (%)	n (%)	Unadjusted HR (95% CI, *p*)^1)^	Age- and gender adjusted HR (95% CI, *p*)^1)^	Multivariate adjusted HR (95% CI, *p*)^1), 4)^
Myocardial infarction or death^2)^	682 (30.7)	1480 (29.6)	0.9 (0.9–1.0, 0,19)	0.9 (0.8–1.0, 0,02)	0.9 (0.8–1.0, 0,05)
Death^3)^	473 (21.3)	938 (18.8)	0.9 (0.8–1.0, 0,005)	0.8 (0.7–0.9, < 0,001)	0.8 (0.7–0.9, < 0,001)

^1)^ Reference: 0–2 preventive treatment targets attained

^2)^ Median follow-up time: 837 days (25th, 75th percentile: 449, 1187)

^3)^ Median follow-up time 974 days (25th, 75th percentile: 578, 1400)

^4)^ Age, gender, heart failure, renal failure and coronary angiogram

## Discussion

This nationwide study showed that prior CAD was frequent (25%) in consecutive patients admitted with acute MI to hospitals in Norway. On average, only half the treatment targets for secondary preventive therapy were attained, and only 1% of patients with MI and prior CAD had reached all the targets. The risk of recurrent MI and death during long-term follow-up was significantly increased in patients with prior CAD compared to patients without prior CAD.

There is compelling scientific evidence supporting that a healthier lifestyle and use of cardioprotective medication in patients with established CAD reduce the risk of recurrent cardiovascular events. Smoking cessation after MI reduces coronary mortality by 36%, and the use of statins reduces the five-year incidence of cardiovascular events by approximately 20% per mmol/L of LDL cholesterol reduction [20, 21]. However, EUROASPIRE IV, a cross-sectional survey from selected centres in 24 European countries, concluded that the majority of coronary patients did not achieve the targets for secondary prevention; six months after acute coronary syndrome or revascularization, 16% of the patients smoked; 38% were obese; 43 and 81% had blood pressure and LDL cholesterol above targets, respectively; and 47% of patients with diabetes did not have their diabetes under control according to the relevant guidelines [13]. Similar findings have been described in other large studies [12, 15, 22, 23]. The present nationwide study from Norway also showing moderate adherence to secondary prevention guidelines in patients with prior CAD admitted with acute MIs, are in accordance with these results. Although Norway has a well-functioning health care service with low direct cost for the patients and most patients received guideline-recommended drugs, control of LDL cholesterol, blood pressure and blood glucose were inadequate. Possible explanations may include a lack of up-titration of drug doses, lack of drug combinations with different modes of actions, and poor patient drug compliance. Another reason might be the lack of a national programme for systematic outpatient secondary prevention follow-up. Such programmes are clearly associated with a reduction in all-cause and cardiac mortality and recurrent cardiovascular events [24]. Less than half the patients included in EUROASPIRE IV accessed a cardiac disease prevention programme [13, 25, 26]. In Norway, there are large differences between hospitals with respect to treatment, prevention programmes and degree of follow-up [27]. Unfortunately, the NORMI does not register information about patients after the primary hospital stay.

Improved survival after MI have been reported in many European countries over the last decades [1]. However, this study demonstrated a high rate of subsequent cardiovascular events after MI in patients with prior CAD. Similar findings have recently been reported from Sweden [2]. These Scandinavian studies both included nationwide populations and had minimal selections bias. Prior CAD was demonstrated to be an independent risk factor for new ischaemic events and death in patients with acute MI [2]. In our opinion, the adverse outcome after MI in patients with prior CAD may partly be explained by the unsatisfactory risk factor control and less invasive therapy of the index MI in patients with prior CAD. The reasons for fewer patients with prior CAD undergoing coronary angiograms and PCI than patients with MI without prior CAD are not clear, but a higher incidence of comorbidities and, consequently, a greater risk of complications, may have been an important factor.

The main strengths of this study are the large and unselected population comprising nearly all patients hospitalized with MI in Norway from 2013 to 2016 and nearly complete follow-up with respect to new MIs and/or death. However, there are some important limitations. This study was an observational study, making it impossible to demonstrate causal associations between prior CAD and long-term outcomes or between the degree of adherence to secondary preventive guidelines and outcomes. Only MIs that led to hospitalization were registered in the NORMI. A few hospitals did not deliver complete data for the whole period, the NORMI did not have complete coverage for all variables e.g. BMI, LDL cholesterol and HbA1c, and the recorded blood pressures were not obtained in a standardized setting. Data on patients transferred between hospitals were merged in the register. This led to some uncertainty, particularly in cases of missing or different registration of the same variable. Nevertheless, the coverage of NORMI compared to the Norwegian Patient Register was good and the degree of completeness and correctness of most variables was high [16, 18]. The NORMI did not register information about patients after the primary hospital stay, and we had no information regarding cardiac rehabilitation programs and causes of death in patients who died.

## Conclusions

One quarter of patients admitted to hospitals in Norway with acute MI had prior CAD. In those patients, the risk factor control was not optimal, and the rates of recurrent MI and death were significantly higher than in patients without prior CAD. In our opinion, a new approach to secondary prevention is needed; all patients with CAD capable of participating in a systematic preventive cardiology programme, should be offered this in order to improve risk factor control.

## Abbreviations

BMI: Body mass index
CABG: Coronary artery bypass grafting
CAD: Coronary artery disease
DAPT: Dual antiplatelet therapy
MI: Myocardial infarction
NORMI: Norwegian myocardial infarction register
PCI: Percutaneous coronary intervention
